# Ribosome Stalling of *N*-Linked Glycoproteins in Cell-Free Extracts

**DOI:** 10.1021/acssynbio.2c00311

**Published:** 2022-11-18

**Authors:** Sean S. Chung, Erik J. Bidstrup, Jasmine M. Hershewe, Katherine F. Warfel, Michael C. Jewett, Matthew P. DeLisa

**Affiliations:** † Biochemistry, Molecular and Cell Biology, Cornell University, Ithaca, New York 14853, United States; ‡ Robert F. Smith School of Chemical and Biomolecular Engineering, Cornell University, Ithaca, New York 14853, United States; § Department of Chemical and Biological Engineering, Northwestern University, 2145 Sheridan Road Technological Institute E136, Evanston, Illinois 60208-3120, United States; ∥ Center for Synthetic Biology, Northwestern University, 2145 Sheridan Road Technological Institute E136, Evanston, Illinois 60208-3120, United States; ⊥ Chemistry of Life Processes Institute, Northwestern University, 2170 Campus Drive, Evanston, Illinois 60208-3120, United States; # Cornell Institute of Biotechnology, Cornell University, Ithaca, New York 14853, United States

**Keywords:** asparagine-linked glycosylation, cell-free protein synthesis, directed evolution, protein display technology, protein engineering, synthetic glycobiology

## Abstract

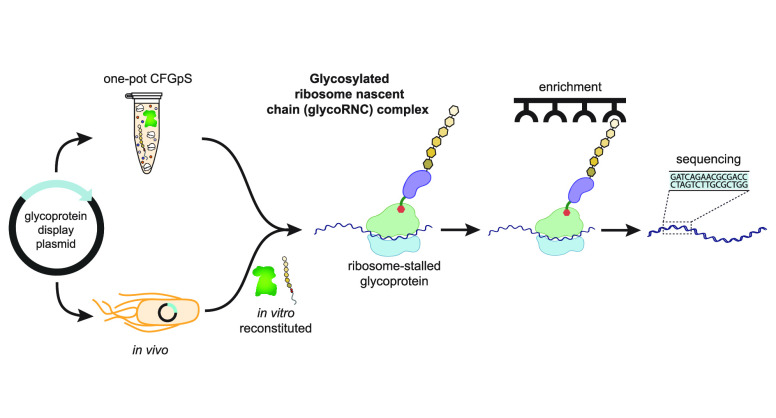

Ribosome display is a powerful *in vitro* method for selection and directed evolution of proteins expressed from combinatorial libraries. However, the ability to display proteins with complex post-translational modifications such as glycosylation is limited. To address this gap, we developed a set of complementary methods for producing stalled ribosome complexes that displayed asparagine-linked (*N*-linked) glycoproteins in conformations amenable to downstream functional and glycostructural interrogation. The ability to generate glycosylated ribosome–nascent chain (glycoRNC) complexes was enabled by integrating SecM-mediated translation arrest with methods for cell-free *N*-glycoprotein synthesis. This integration enabled a first-in-kind method for ribosome stalling of target proteins modified efficiently and site-specifically with different *N*-glycan structures. Moreover, the observation that encoding mRNAs remained stably attached to ribosomes provides evidence of a genotype–glycophenotype link between an arrested glycoprotein and its RNA message. We anticipate that our method will enable selection and evolution of *N*-glycoproteins with advantageous biological and biophysical properties.

## Introduction

Asparagine-linked (*N*-linked) protein glycosylation, the chemical modification of specific amino acid side chains with oligosaccharides termed glycans, is a conserved co- and post-translational modification (PTM) that occurs in all domains of life.^1^ It is one of the most abundant PTMs in nature and serves to expand the diversity of secretory and membrane proteins. Importantly, *N*-linked glycans are well-known to modulate functional and structural properties of proteins.^2^ The *N*-glycosylation process involves assembly of lipid-linked oligosaccharides (LLOs) bearing *N*-glycan structures that are subsequently transferred from the lipid carrier onto asparagine residues in acceptor proteins by an oligosaccharyltransferase (OST).

A major breakthrough in our ability to study and engineer *N*-glycosylation occurred when Aebi and co-workers functionally transferred the bacterial glycosylation machinery encoded by the *Campylobacter jejunipgl* locus into *Escherichia coli* cells, which do not natively perform protein glycosylation.^3^ Since this early pioneering work, diverse proteins of prokaryotic and eukaryotic origin have been *N*-glycosylated in engineered *E. coli* cells carrying the *pgl* glycosylation pathway^4−6^ or other heterologous glycosylation pathways.^7,8^ More recently, glycoengineered *E. coli* have been leveraged as source strains to provide cell-free extracts selectively enriched with protein glycosylation machinery, including OSTs and LLOs.^9−12^ Upon addition of cofactors and plasmid DNA encoding an acceptor protein of interest, these glyco-enriched cell-free extracts enable a one-pot reaction scheme for site-specific expression and glycosylation of target glycoproteins at relatively high titers.

With the advent of *E. coli* cell-based and cell-free methods for customizable protein glycosylation, it becomes possible to develop advanced peptide/protein display techniques such as phage, ribosome, and mRNA display for high-throughput screening of *N*-glycoprotein libraries. One representative example is the extension of phage display to include *N*-linked glycosylation based on the *C. jejunipgl* system.^13^ A key feature of this “glycophage” technology is the establishment of a physical coupling between the phenotype of the displayed glycoprotein (glycophenotype) and the corresponding genotype encoded in the phage genome. The resulting genotype–glycophenotype link was subsequently leveraged to select functional glycosylation sequons from libraries of randomized acceptor sequences.^13^

Protein display methods that are entirely cell-free, such as ribosome and mRNA display^14^, are attractive alternatives to phage display because they potentially enable larger libraries (>10^9^) and greater diversity due to DNA library transcription occurring *in vitro*. However, while cell-free display methods have been widely used with great success, they have yet to be extended to *N*-linked glycoproteins with natural glycosidic bonds. This technology gap is most likely due to the fact that conventional *in vitro* translation systems, on which these methods depend for producing displayed proteins, are limited by their inability to coactivate efficient protein synthesis and glycosylation. For example, cell-free protein synthesis (CFPS) systems based on *E. coli* (*e.g.*, S30 extract, PURE system) are incapable of making glycoproteins because these systems lack glycosylation machinery.^15^ Other commonly used eukaryotic CFPS systems (*e.g.*, rabbit reticulocyte, wheat germ extracts) also cannot perform glycosylation because they lack microsomes.^16^ While glycosylation in eukaryotic CFPS systems can be introduced *via* microsome supplementation or enrichment,^17^ the resulting glycoprotein yields in these systems are often low due to the poor compatibility between the extract translational machinery and microsomal glycosylation machinery. Protein glycosylation in these microsomal vesicles is difficult to control or engineer and likely incompatible with protein display on ribosomes or mRNA due to the sequestration of glycoproteins inside the vesicles. Indeed, the glycophage display method described above was possible only because the processes of phage assembly and *N*-linked protein glycosylation could be harmonized in the periplasm of living *E. coli* cells.

In this study, we investigated the extent to which ribosome display is compatible with emerging methods for cell-free glycoprotein biosynthesis (CFGpS) based on extracts derived from glycoengineered *E. coli* that effectively couple transcription/translation with glycosylation.^9,10^ A prerequisite for selecting proteins from ribosome display libraries is the genotype–phenotype link, which is accomplished during *in vitro* translation by stabilizing a complex consisting of the ribosome, the mRNA, and the nascent, correctly folded polypeptide. To generate this crucial genotype–phenotype link, we combined two different cell-free protein glycosylation strategies with the 17 amino acid ribosome stall sequence (FSTPVWISQAQGIRAGP) derived from *E. coli* SecM (SecM17)^18^ that enables long-lived ribosome stalling of heterologous proteins of interest (POIs) and their encoding mRNAs in living *E. coli* cells and cell-free extracts.^19,20^ The resulting methods enabled production of glycosylated ribosome–nascent chain (glycoRNC) complexes that displayed several different *N*-linked glycoproteins in conformations that were sufficiently exposed to permit downstream functional and glycostructural interrogation. Importantly, mRNA transcripts encoding the displayed glycoproteins were found stably attached to stalled ribosomes both before and after biopanning, thereby providing the crucial physical linkage between an arrested glycoprotein and its RNA transcript, paving the way for future selection and evolution of *N*-linked glycoproteins with desirable properties.

## Results and Discussion

**Figure 1:**
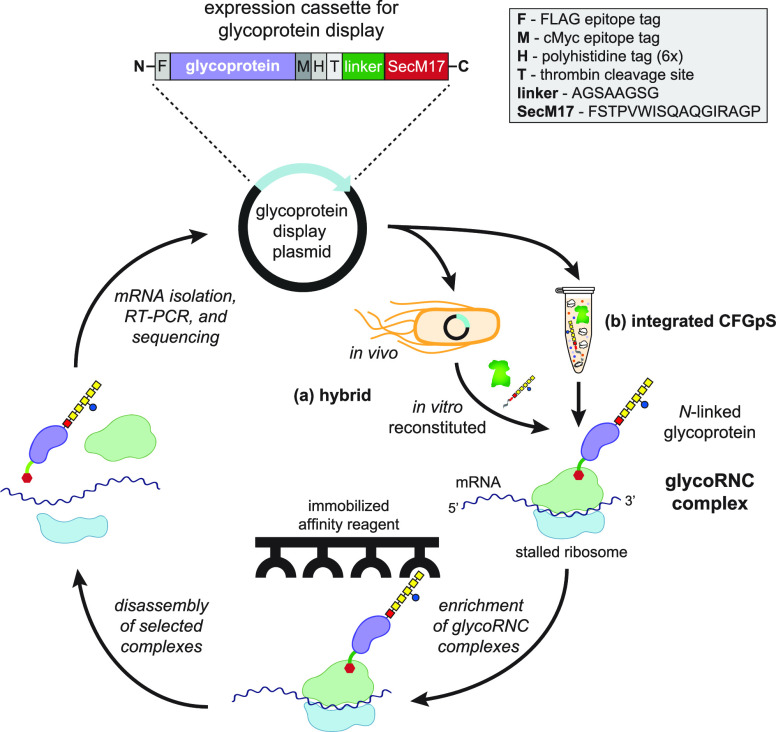
Cell-free glycoprotein display on ribosomes. A schematic of ribosome display for *N*-linked glycoproteins is shown. In the hybrid *in vivo*–*in vitro* approach, living *E. coli* cells are transformed with a plasmid for expressing glycoPOI–SecM17 fusions. Following 70S ribosome isolation, *N*-glycan installation on ribosome-stalled glycoproteins is performed in a subsequent *in vitro* glycosylation step. In the integrated CFGpS approach, plasmid DNA is used to prime a reaction mixture in which transcription, translation, and *N*-linked glycosylation occur in a single pot. Both approaches yield glycoRNC complexes, which are subjected to function-based affinity selection (*i.e.*, binding to immobilized antigen) and/or glycosylation-based affinity selection (*i.e.*, binding to immobilized antibody or lectin specific for the *N*-glycan). Bound complexes are dissociated by EDTA or specifically eluted with antigen or glycan, and the identities of bound glycoproteins are determined by sequencing RT-PCR products. This image was created with BioRender.

Ribosome display of *N*-linked glycoproteins hinges on the generation of glycoRNC complexes (Figure 1). Therefore, we first investigated whether target acceptor proteins were amenable to *N*-linked glycosylation poststalling using a hybrid *in vivo*–*in vitro* approach. For the *in-vivo* step, expression of a POI–SecM17 fusion was performed in living *E. coli* cells from which 70S ribosomes were subsequently isolated. For the *in vitro* step, isolated 70S ribosomes were subjected to an *in vitro* reconstituted glycosylation system to install *N*-glycans on the SecM17-arrested POI. Importantly, we used a previously identified linker sequence between the POI and SecM17 that had sufficient length to fully expose stalled proteins outside the ribosome exit tunnel, rendering them available for functional interrogation.^19^ Our initial POI was the *E. coli* immunity protein, Im7, a globular 87-residue protein that is well-expressed in *E. coli*. While not a native glycoprotein, Im7 has been shown to tolerate *N*-glycan installation at numerous artificial DQNAT acceptor sites throughout its structure using both cellular and cell-free glycosylation systems based on the *C. jejunipgl* machinery.^6^ We specifically chose the Im7^N58^ mutant (where the superscript denotes the location of the asparagine residue), as it is efficiently glycosylated *in vitro*.^6^

To confirm ribosome stalling of Im7^N58^–SecM17 fusions, we first isolated 70S ribosomes from *E. coli* cell lysates *via* sucrose cushion centrifugation. Western blot analysis demonstrated that only the Im7^N58^–SecM17 fusion but not Im7^N58^ lacking the SecM17 stall sequence was present in ribosome fractions, as judged by the absorbance profile at 254 nm (*A*_254_) and the sedimentation position (70S) (Supplementary Figure 1a). These results confirmed that coelution of Im7^N58^ with ribosomes depended on the SecM17 stall signal. Arrested expression of Im7^N58^–SecM17 on ribosomes did not significantly affect ribosomal composition, as evidenced by the similar protein profiles for ribosome preparations derived from cells expressing Im7^N58^ with or without SecM17 (Supplementary Figure 1b). Likewise, the growth rates of cells expressing Im7^N58^ with or without SecM17 during the induction period were indistinguishable relative to cells containing an empty expression vector (data not shown).

**Figure 2:**
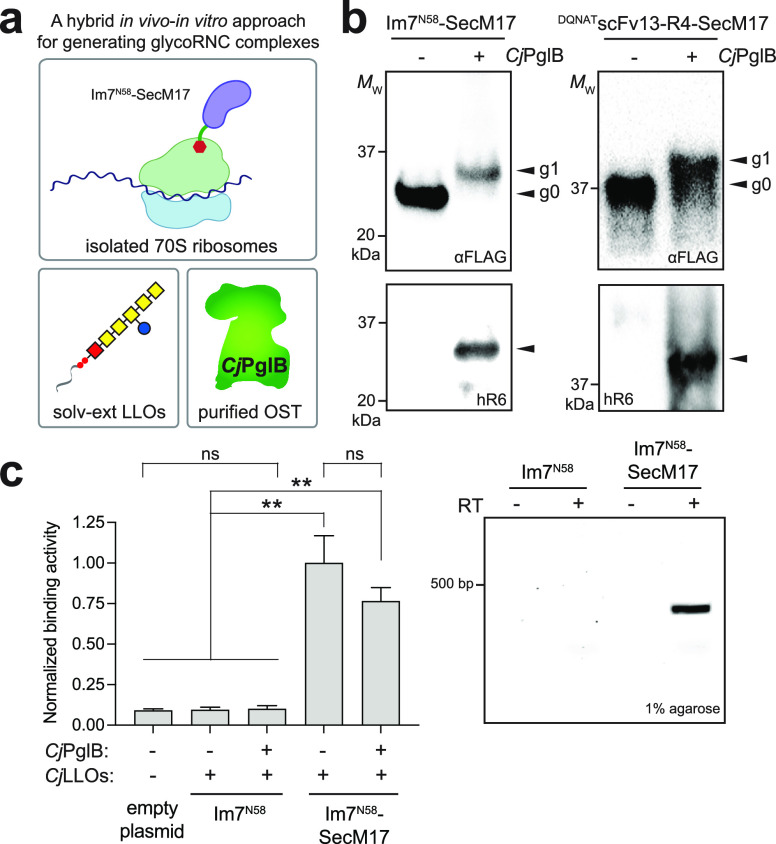
Hybrid *in vivo*–*in vitro* strategy for generating and selecting glycoRNC complexes. (a) Schematic of hybrid strategy in which 70S ribosomes derived from living cells (*in vivo*) are isolated and subjected to cell-free glycosylation (*in vitro*). This image was created with BioRender. (b) Western blot analysis of 70S ribosome fractions derived from cells expressing Im7^N58^–SecM17 or ^DQNAT^scFv13-R4–SecM17 and subjected to *in vitro* glycosylation with (+) or without (−) *Cj*PglB along with solvent-extracted *Cj*LLOs. Blots were probed with anti-FLAG antibody to detect acceptor protein (top) and hR6 serum against the glycan (bottom). Markers for aglycosylated (g0) and singly glycosylated (g1) forms of acceptor protein are indicated at the right. Molecular weight (*M*_W_) markers are indicated at the left. Blots are representative of biological replicates (*n* = 2). (c) ColE7-binding activity measured for 70S ribosomes isolated from cells expressing Im7^N58^ or Im7^N58^–SecM17 with (+) or without (−) *Cj*PglB along with solvent-extracted *Cj*LLOs. 70S ribosomes from cells carrying empty plasmid served as a negative control. Data are averages of biological replicates (*n* = 3) ± SD. Statistical significance was determined by unpaired *t* test with Welch’s correction (*, *p* < 0.05; **, *p* < 0.01; ns, not significant). (d) Postselection detection of mRNA associated with ribosomes enriched *via* ColE7 biopanning. mRNA isolated from functionally selected ribosomes was subjected to RT-PCR with (+) or without (−) reverse transcriptase (RT). Results are representative of biological replicates (*n* = 2).

To create glycoRNC complexes, we hypothesized that 70S ribosome preparations derived from the *in vivo* step above could be used as purified acceptor substrates for an *in vitro* reconstituted glycosylation assay. To test this notion, RNC complexes displaying aglycosylated Im7^N58^–SecM17 were combined with purified PglB OST from *C. jejuni* (*Cj*PglB) and solvent-extracted LLOs bearing the *C. jejuniN*-glycan (*Cj*LLOs) (Figure 2a). Western blot analysis of the *in vitro* glycosylation reaction products was performed using an anti-FLAG antibody to detect the Im7^N58^–SecM17 protein and hR6 serum to specifically detect the *C. jejuniN*-glycan.^21^ A shift in the apparent molecular weight of Im7^N58^–SecM17 in the anti-FLAG immunoblot that corresponded to a similarly sized band detected in the hR6 immunoblot confirmed that ribosome-tethered Im7^N58^–SecM17 was nearly 100% glycosylated (Figure 2b). When *Cj*PglB was omitted from the reaction, we observed no detectable glycosylation of ribosome-stalled Im7^N58^–SecM17, confirming that glycosylation of ribosome-stalled acceptor proteins was OST-dependent.

To demonstrate the modularity of our hybrid approach, we swapped the POI from Im7^N58^ to a single-chain Fv antibody specific for β-galactosidase modified with a DQNAT glycosylation tag at its N-terminus (^DQNAT^scFv13-R4).^22^ Of note, previous studies confirmed that scFv13-R4 bearing a C-terminal DQNAT tag was glycosylated by the *C. jejuni* glycosylation machinery.^4,9^ Here the ^DQNAT^scFv13-R4–SecM17 construct was present in sucrose gradient fractions containing 70S ribosomes based on the *A*_254_ profile, sedimentation position, and Western blot analysis of the resulting sucrose gradient fractions (Supplementary Figure 2). Similar to Im7^N58^–SecM17, ^DQNAT^scFv13–R4-SecM17 was efficiently glycosylated in a *Cj*PglB-dependent manner (Figure 2b), leading to the formation of glycoRNC complexes displaying the glycosylated scFv antibody. We next determined whether intact mRNA encoding the POI remained associated with glycoRNC complexes following functional selection. Specifically, a biopanning procedure was performed in which *in vitro* glycosylation reaction products were directly incubated in enzyme-linked immunosorbent assay (ELISA) plates coated with ColE7, a 60 kDa bacterial toxin that is inhibited by Im7 binding.^23^ Following extensive washing, strong antigen-binding activity was measured for stalled RNC complexes displaying glycosylated Im7^N58^–SecM17 that was comparable to that observed for aglycosylated Im7^N58^–SecM17 (Figure 2c), indicating that ribosome stalling of glycosylated Im7^N58^ was compatible with ColE7-binding activity. Upon verification of affinity capture, the bound glycoRNC complexes were dissociated with EDTA, and the ribosome-associated RNA, including stalled mRNA, was isolated from dissociated complexes. To detect the presence of mRNA, reverse transcription polymerase chain reaction (RT-PCR) was performed using primers specific for the Im7^N58^ gene sequence. By this method, we determined that RNA recovered from functionally selected glycoRNC complexes gave rise to clearly detectable amplicons corresponding in size to the full-length Im7^N58^ sequence (Figure 2d), which was confirmed by sequencing. In contrast, RT-PCR performed on RNA recovered from samples corresponding to Im7^58^ lacking the SecM17 stall sequence produced no detectable amplicons, indicating that the SecM17 stall sequence is essential for functional selection of glycoRNC complexes. Collectively, these data confirmed that the model Im7^N58^ glycoprotein and its encoding mRNA were stably attached to stalled ribosomes, thereby creating the essential genotype–glycophenotype link required for library screening.

**Figure 3:**
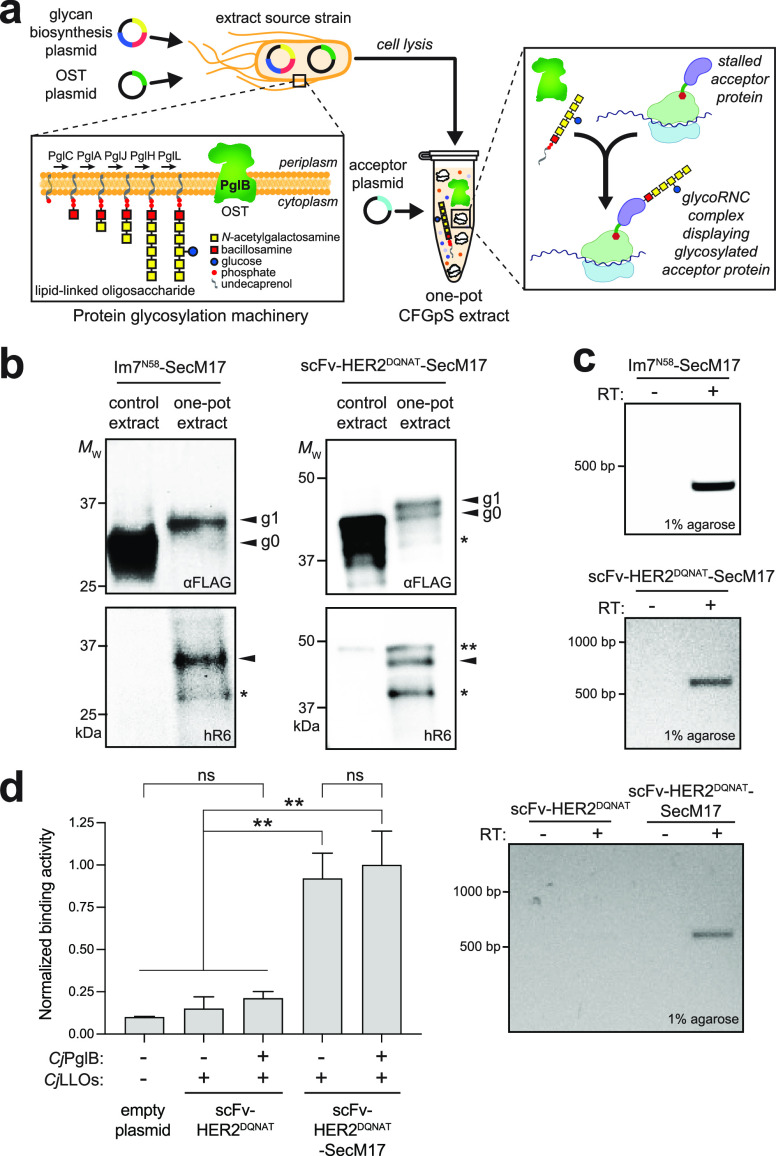
One-pot cell-free strategy for generating and selecting glycoRNC complexes. (a) Schematic of the one-pot strategy in which 70S ribosomes are derived from glycoenriched CFGpS extracts. This image was created with BioRender. (b) Western blot analysis of 70S ribosome fractions derived from CFGpS extracts coenriched with *Cj*PglB and *Cj*LLOs (one-pot) or enriched with *Cj*LLOs only (control) that were primed with plasmids encoding Im7^N58^–SecM17 or scFv-HER2^DQNAT^–SecM17. Blots were probed with anti-FLAG antibody to detect acceptor protein (top) and hR6 serum against the glycan (bottom). Markers for aglycosylated (g0) and singly glycosylated (g1) forms of acceptor proteins are indicated at the right. The asterisk (*) denotes the degradation product, and the double asterisk (**) denotes the nonspecific product. Molecular weight (*M*_W_) markers are indicated at the left. Blots are representative of biological replicates (*n* = 2). (c) Preselection detection of mRNA associated with 70S ribosomes displaying glycosylated Im7^N58^–SecM17 or scFv-HER2^DQNAT^–SecM17. RT-PCR was performed with (+) or without (−) RT. (d, left) HER2-binding activity measured for 70S ribosomes isolated from CFGpS reactions primed with plasmid DNA encoding scFv-HER2^DQNAT^ or scFv-HER2^DQNAT^–SecM17 with (+) or without (−) *Cj*PglB. 70S ribosomes from cells carrying empty plasmid served as the negative control. Data are averages of biological replicates (*n* = 3) ± SD. Statistical significance was determined by unpaired *t* test with Welch’s correction (*, *p* < 0.05; **, *p* < 0.01; ns, not significant). (d, right) Postselection detection of mRNA associated with ribosomes enriched *via* HER2 biopanning. mRNA isolated from functionally selected ribosomes was subjected to RT-PCR in the presence (+) or absence (−) of RT. Results are representative of biological replicates (*n* = 2).

We also investigated an alternative strategy for generating glycoRNC complexes *via* an entirely cell-free procedure that circumvented the need for *in vivo* expression and purification of stalled 70S ribosome–POI complexes and supplementation of laboriously extracted/purified glycosylation components. We hypothesized that such an approach would be possible by integrating SecM-mediated translation arrest with a previously described CFGpS technology in which transcription and translation are coupled to *N*-linked glycosylation in a single pot^9^ (Figure 3a). Using Im7^N58^ as the POI, we generated a cell-free expression plasmid in which this acceptor protein was fused to the SecM17 stall sequence. In parallel, glycoengineered *E. coli* CLM24 cells were used to prepare S12 extracts that were selectively enriched with both *Cj*PglB and *Cj*LLOs.^9^ Our previous work showed that S12 extract can achieve higher glycosylation efficiencies than S30 extract due to its increased concentration of vesicle-bound glycosylation machinery.^11^ To generate glycoRNC complexes, batch-mode sequential CFGpS reactions were performed by priming glycoenriched extracts with plasmid DNA encoding Im7^N58^–SecM17 followed by isolation of 70S ribosomes by sucrose cushion centrifugation. Western blot analysis of ribosome fractions with anti-FLAG antibody and hR6 serum revealed efficient glycosylation of Im7^N58^–SecM17 on stalled ribosomes, with nearly 100% of the acceptor protein appearing as the g1 form in the anti-FLAG blot (Figure 3b). When S12 extracts were enriched with only *Cj*LLOs but not *Cj*PglB, there was no detectable glycosylation of Im7^N58^–SecM17 on stalled ribosomes. Importantly, the mRNA encoding SecM17-stalled Im7^N58^ was stably attached to ribosomes, as evidenced by the prominent amplicon generated *via* RT-PCR of ribosome-associated RNA (Figure 3c). Nearly identical results were obtained when CFGpS reactions were primed with plasmid DNA encoding a different POI, namely, an scFv antibody specific for human epidermal growth factor receptor 2 (scFv-HER2) that was modified at its C-terminus with a DQNAT glycosylation tag^6,24^ (Figure 3b,c). Using immobilized HER2 as the antigen, we selected glycoRNC complexes displaying glycosylated scFv-HER2^DQNAT^–SecM17 and observed that the encoding mRNA remained associated with these functionally selected glycoRNCs (Figure 3d). In contrast, no mRNA was detected for ribosome complexes prepared from CFGpS reactions primed with plasmid DNA encoding unfused scFv-HER2^DQNAT^, indicating that functional selection of glycoRNCs was dependent on SecM17.

**Figure 4:**
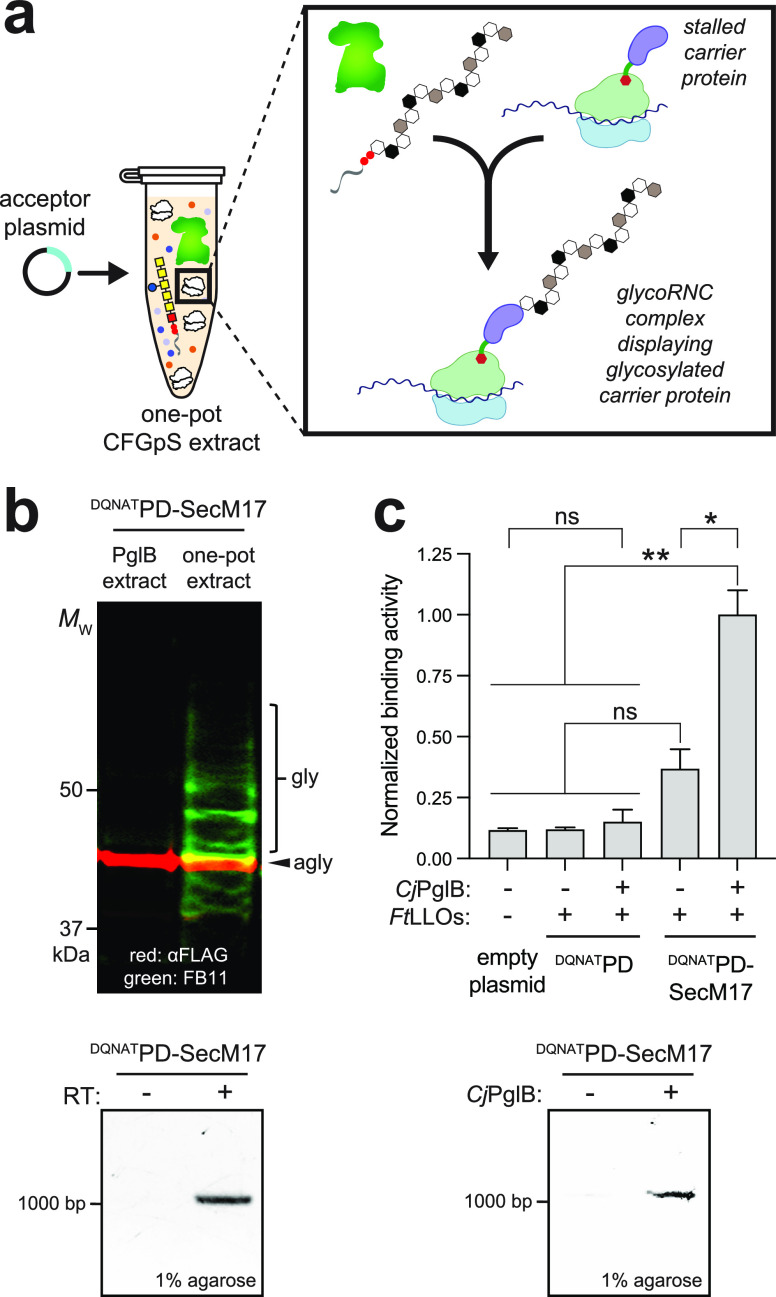
Display and selection of ribosome-stalled bioconjugate vaccine. (a) Schematic of the one-pot strategy for producing 70S ribosomes that display glycosylated vaccine carrier proteins. This image was created with BioRender. (b, top) Western blot analysis of 70S ribosome fractions derived from CFGpS extracts coenriched with *Cj*PglB and *Ft*LLOs (one-pot) or enriched with only *Ft*LLOs (control) that were primed with plasmid DNA encoding ^DQNAT^PD–SecM17. A merged image of blots that were probed with anti-FLAG antibody to detect the acceptor protein (red) and hR6 serum against the glycan (green) is shown. Markers for aglycosylated (agly) and multiply glycosylated (gly) forms of acceptor proteins are indicated at the right. Western blots are representative of biological replicates (*n* = 2). (b, bottom) Preselection detection of mRNA associated with 70S ribosomes displaying glycosylated ^DQNAT^PD–SecM17 with (+) or without (−) RT. (c, top) FB11-binding activity measured for 70S ribosomes isolated from CFGpS reactions primed with plasmid DNA encoding ^DQNAT^PD–SecM17 with (+) or without (−) *Cj*PglB. 70S ribosomes derived from cells carrying empty plasmid served as negative control. Data are averages of biological replicates (*n* = 3) ± SD. Statistical significance was determined by unpaired *t* test with Welch’s correction (*, *p* < 0.05; **, *p* < 0.01; ns, not significant). (c, bottom) Postselection detection of mRNA associated with ribosomes enriched *viaFt*O-PS-specific biopanning with FB11 antibody. mRNA was isolated from selected ribosomes corresponding to glycosylated (+) or aglycosylated (−) ^DQNAT^PD–SecM17 and subjected to RT-PCR. Results are representative of biological replicates (*n* = 2).

To expand the utility of our glycoprotein display method, we designed a one-pot cell-free bioconjugation strategy for displaying conjugate vaccine candidates on 70S ribosomes (Figure 4a). Cell-free bioconjugation is a recently described approach that leverages glycocompetent cell-free extracts for producing conjugate vaccines that retain native immunogenic structures.^12^ In this approach, cell-free extracts are coenriched with an OST and LLOs bearing heterologously expressed capsular polysaccharides (CPSs) or O-antigen polysaccharides (O-PSs). The glycoenriched extracts are then primed with plasmid DNA encoding a vaccine carrier protein such as non-acylated *Haemophilus influenzae* protein D (PD), yielding product proteins that are site-specifically glycosylated with CPS or O-PS antigens of interest.

Here we integrated this cell-free vaccine expression technology with SecM-mediated stalling to engineer glycoRNC complexes that displayed a vaccine carrier protein modified with an O-PS antigen. We focused on PD because it has been shown to be a safe and effective conjugate vaccine carrier protein^25^ that can be efficiently glycosylated with the *Francisella tularensis* O-PS (*Ft*O-PS) antigen in CFGpS.^12^ Here PD was modified at its N-terminus with a DQNAT acceptor motif and at its C-terminus with the SecM17 stall sequence. In parallel, glycoengineered *E. coli* CLM24 cells were used to prepare S12 extracts that were selectively enriched with *Cj*PglB and LLOs bearing the *Ft*O-PS antigen. Our previous work showed that such extracts are selectively enriched with these components.^12^ Next, batch-mode sequential CFGpS reactions were performed by priming these glycoenriched extracts with plasmid DNA encoding the ^DQNAT^PD–SecM17 construct. Isolation of 70S ribosomes and Western blot analysis were performed as described above but with antibody FB11, which specifically recognizes the *Ft*O-PS antigen.^26^ Importantly, the CFGpS reaction mixture promoted efficient glycosylation of ^DQNAT^PD-SecM17 on stalled ribosomes with a ladderlike banding pattern (Figure 4b), which was similar to laddering seen previously for *Ft*O-PS conjugates and arises from variable O-PS chain lengths generated by the Wzy polymerase.^12^ As above, the encoding mRNA was stably associated with glycoRNC complexes displaying stalled ^DQNAT^PD–SecM17 (Figure 4b). We also demonstrated glycan-based selection of RNC complexes displaying glycosylated ^DQNAT^PD–SecM17 using immobilized FB11 antibody, which preferentially enriched RNC complexes bearing the *Ft*O-PS antigen over aglycosylated RNC complexes or empty ribosomes corresponding to ^DQNAT^PD that lacked the SecM17 stall sequence (Figure 4c). Importantly, the encoding mRNA remained associated with the enriched glycoRNCs (Figure 4c), indicating that ribosome-stalled conjugates could be directly selected based on their glyco-phenotype. In contrast, only a faint mRNA signal was detected for ribosomes prepared from CFGpS extracts that lacked *Cj*PglB, confirming that glycan-based enrichment had occurred.

Collectively, the results of our study demonstrate that ribosome stalling is compatible with emerging methods for cell-free glycoprotein biosynthesis (CFGpS),^9,10^ enabling the display of several different *N*-linked glycoproteins on ribosomes in a manner that is compatible with downstream functional and glycostructural interrogation. While our work represents the first demonstration of ribosome stalling of *N*-linked glycoproteins, we note that phage display has previously been extended for displaying post-translationally modified proteins, including *N*-glycoproteins^13^ as well as phosphorylated and phosphopantetheinylated protein.^27^ A handful of reports have also described the use of phage or mRNA to display short peptides (5–33 residues) that were chemically modified by either a single mannose or a high-mannose glycan.^28−30^ In each case, the attachment was through a non-natural linkage, including formation of a disulfide bond between the side chain of a cysteine residue and 2-(3-nitropyridyl disulfide ethyl)mannopyranoside (Man-Npys),^28^ oxime ligation following oxidation of an N-terminal Ser/Thr residue,^29^ and installation of the unnatural amino acid homopropargylglycine that was “click”-glycosylated with Man_9_-azide through copper-assisted azide–alkyne cycloaddition (CuAAAC).^30^ In contrast, our method involved more natural *N*-glycan installation *via* OST-catalyzed glycosidic bond formation between the reducing-end sugar and the side chain of an asparagine residue.

An important facet of this work was the successful creation of a physical link between the ribosome-stalled glycoprotein and its encoding mRNA. Although not directly demonstrated here, this linkage will be crucial for future efforts focused on uncovering the functional and structural consequences of *N*-glycan installation as well as the selection and evolution of *N*-linked glycoproteins with desirable properties. The utility of our method for such applications will depend in part on whether the two underlying cell-free glycosylation strategies can install glycans onto acceptor sites that are located at internal locations within structurally complex proteins. In the present work, we showed glycosylation at an internal site engineered within Im7, which provides preliminary evidence for the ability to glycosylate proteins beyond easily accessible N- or C-terminal glycosylation tags. Our previous studies demonstrated that cell-free glycosylation methods could install *N*-linked glycans at three native glycosylation sites located internally within human erythropoietin (EPO)^9^ and at a variety of bespoke locations throughout the structure of bovine RNase A.^6^ These examples illustrate the ability of different cell-free glycosylation strategies to introduce glycans into complex protein structures *in situ*.

Display of *N*-linked glycoproteins on ribosomes stands alongside a growing list of screening and selection tools that are amenable to glycoprotein engineering. Besides the glycophage display methods discussed above, several other high-throughput genetic assays for *N*-linked glycosylation have been described including enzyme-linked immunosorbent assay (ELISA)-based detection of periplasmic *N*-glycoproteins,^31^ cell-surface display of *N*-glycans and *N*-glycoproteins,^5,8^ and a colony replica blotting strategy called glycosylation of secreted *N*-linked acceptor proteins (glycoSNAP).^4,6^ Collectively, these assays are enabling the creation and evaluation of an unprecedentedly large number of intact glycoproteins (>150 in one study alone^6^) for which the structure–activity relationships associated with *N*-glycan installation can be systematically catalogued or technologically exploited. Indeed, our demonstration of ribosome stalling for a conjugate vaccine candidate composed of an FDA-approved carrier protein and a pathogen-specific polysaccharide paves the way for studying and engineering the biological, biophysical, and immunological properties of this important class of new-to-nature glycoproteins.

## Materials and Methods

### Bacterial Strains and Plasmids

*E. coli* strain DH5α was used for cloning and maintenance of plasmids. *E. coli* strain BL21(DE3) was used for *in vivo* production of RNC complexes displaying different POIs and for production of ColE7^H569A^ as described previously.^6^*E. coli* strain CLM24 was used for purification of the *Cj*PglB enzyme and organic-solvent-based extraction of *Cj*LLOs used for *in vitro* glycosylation as described previously.^9^ CLM24 was also used as the source strain for preparing cell-free extracts with selectively enriched glycosylation components as described previously.^9^ CLM24 is a glyco-optimized derivative of W3110 that carries a deletion in the gene encoding the WaaL ligase, thus facilitating the accumulation of preassembled glycans on undecaprenyl phosphate.^7^ All of the plasmids used in this study, including new plasmid constructions, are described in Supplementary Methods

### Preparation of Ribosomes, OSTs, LLOs, and Immobilized Antigens

Ribosomes were isolated from soluble cell fractions or cell-free extracts using sucrose cushion centrifugation according to previously published procedures.^19,20^ Purification of *Cj*PglB and organic solvent extraction of *Cj*LLOs from *E. coli* membranes were both performed according to previously described protocols.^15^ Purification of ColE7 was performed according to standard Ni-NTA affinity purification. Each of these procedures is described in detail in Supplementary Methods.

### Preparation of Crude S12 Extracts

CLM24 source strains were grown in 2× YTP (10 g/L yeast extract, 16 g/L tryptone, 5 g/L NaCl, 7 g/L K_2_HPO_4_, 3 g/L KH_2_PO_4_, pH 7.2). To generate *Cj*LLO-enriched extract, CLM24 carrying plasmid^32^ pMW07–pglΔ*B* for the *C. jejuni* glycan or pGAB2 for the *Ft*O-PS^##^ was used as the source strain. To generate one-pot extracts containing both *Cj*PglB and *Cj*LLOs, CLM24 carrying pMW07–pglΔ*B* and pSF–*Cj*PglB^932^^32^ was used as the source strain. After inoculation, the expression of *Cj*PglB was induced at an Abs_600_ of 0.8 with l-arabinose to a final concentration of 0.2% w/v. After induction, protein expression was allowed to proceed at 30 °C to a density of Abs_600_ ∼ 3, at which point cells were harvested by centrifugation (5000*g*) at 4 °C for 15 min. All subsequent steps were carried out at 4 °C unless otherwise stated. Cells were harvested and washed twice using S12 buffer (10 mM Tris acetate, 14 mM magnesium acetate, 60 mM potassium acetate, pH 8.2). The pellet was then resuspended in 1 mL of S12 buffer per 1 g of cells. The resulting suspension was passed once through a EmulsiFlex-B15 high-pressure homogenizer (Avestin) at 20,000–25,000 psi to lyse cells. The extract was then centrifuged at 12,000*g* for 10 min to remove cell debris, and the supernatant was collected and incubated at 37 °C for 60 min with shaking at 250 rpm for the runoff reaction. Following centrifugation at 10,000*g* for 10 min at 4 °C, the supernatant was collected, flash-frozen in liquid nitrogen, and stored at −80 °C.

### Cell-Free Glycosylation

For *in vitro* reconstituted glycosylation of *E. coli* cell-derived RNC complexes displaying Im7^N58^ or ^DQNAT^scFv13-R4, reactions were carried out in a 50 μL volume containing 3 μg of ribosome-stalled acceptor protein, 2 μg of purified *Cj*PglB, and 5 μg of extracted LLOs in *in vitro* glycosylation buffer (10 mM HEPES, pH 7.5, 10 mM MnCl_2_, 0.1% w/v DDM). The reaction mixture was incubated at 30 °C for 16 h. For CFGpS, reactions were carried out in 1 mL reaction volumes in a 15 mL conical tube using a modified PANOx-SP system.^33^ The reaction mixture contained the following components: 0.85 mM GTP, 0.85 mM UTP, 0.85 mM CTP, 1.2 mM ATP, 34.0 μg/mL folinic acid, 170.0 μg/mL *E. coli* tRNA mixture, 130 mM potassium glutamate, 10 mM ammonium glutamate, 12 mM magnesium glutamate, 20 amino acids (each at 2 mM), 0.4 mM nicotinamide adenine dinucleotide (NAD), 0.27 mM coenzyme-A (CoA), 1.5 mM spermidine, 1 mM putrescine, 4 mM sodium oxalate, 33 mM phosphoenolpyruvate (PEP), 57 mM HEPES, 6.67 μg/mL plasmid, and 27% v/v cell lysate. Protein synthesis was carried out for 30 min at 30 °C, after which protein glycosylation was initiated by the addition of MnCl_2_ and DDM at a final concentration of 10 mM and 0.1% w/v, respectively, and allowed to proceed at 30 °C for 16 h. Isolation of ribosomes from *in vitro* reconstituted glycosylation and CFGpS reactions was performed as described above.

### Characterization and Affinity Enrichment of GlycoRNCs

Western blot analysis and ELISA were performed according to standard protocols as described in Supplementary Methods. Affinity selection of ribosome complexes, mRNA isolation, and RT-PCR were performed as described previously.^19^ Each of these procedures is described in detail in Supplementary Methods.

### Statistical Analysis and Reproducibility

All data were reported as average values with error bars representing standard deviation. Statistical significance was determined by unpaired *t* test with Welch’s correction (*, *p* < 0.05; **, *p* < 0.01; ns, not significant). All graphs were generated using Prism 9 for MacOS version 9.2.0.

## Supporting Information Available

Detailed description of methods, purification and characterization of 70S ribosomes displaying Im7^N56^-SecM17, and purification and characterization of 70S ribosomes displaying ^DQNAT^scFv13-R4-SecM17 (PDF)

## References

[ref1] Abu-Qarn M.; Eichler J.; Sharon N. Not just for Eukarya anymore: protein glycosylation in Bacteria and Archaea. Curr. Opin. Struct. Biol. 2008, 18 (5), 544–550. 10.1016/j.sbi.2008.06.010. 18694827

[ref2] Varki A.; Gagneux P. Biological Functions of Glycans. In Essentials of Glycobiology, 3rd ed.; Varki A.; Cummings R. D.; Esko J. D.; Stanley P.; Hart G. W.; Aebi M.; Darvill A. G.; Kinoshita T.; Packer N. H.; Prestegard J. H.; Schnaar R. L.; Seeberger P. H., Eds.; Cold Spring Harbor Laboratory Press: Cold Spring Harbor, NY, 2015; pp 77–88.

[ref3] Wacker M.; Linton D.; Hitchen P. G.; Nita-Lazar M.; Haslam S. M.; North S. J.; Panico M.; Morris H. R.; Dell A.; Wren B. W.; et al. *N*-linked glycosylation in *Campylobacter jejuni* and its functional transfer into *E. coli*. Science 2002, 298 (5599), 1790–1793. 10.1126/science.298.5599.1790. 12459590

[ref4] Ollis A. A.; Zhang S.; Fisher A. C.; DeLisa M. P. Engineered oligosaccharyltransferases with greatly relaxed acceptor-site specificity. Nat. Chem. Biol. 2014, 10 (10), 816–822. 10.1038/nchembio.1609. 25129029 PMC4575499

[ref5] Fisher A. C.; Haitjema C. H.; Guarino C.; Celik E.; Endicott C. E.; Reading C. A.; Merritt J. H.; Ptak A. C.; Zhang S.; DeLisa M. P. Production of secretory and extracellular *N*-linked glycoproteins in *Escherichia coli*. Appl. Environ. Microbiol. 2011, 77 (3), 871–881. 10.1128/AEM.01901-10. 21131519 PMC3028701

[ref6] Li M.; Zheng X.; Shanker S.; Jaroentomeechai T.; Moeller T. D.; Hulbert S. W.; Kocer I.; Byrne J.; Cox E. C.; Fu Q. Shotgun scanning glycomutagenesis: A simple and efficient strategy for constructing and characterizing neoglycoproteins. Proc. Natl. Acad. Sci. U. S. A. 2021, 118 (39), e2107440118 10.1073/pnas.2107440118. 34551980 PMC8488656

[ref7] Feldman M. F.; Wacker M.; Hernandez M.; Hitchen P. G.; Marolda C. L.; Kowarik M.; Morris H. R.; Dell A.; Valvano M. A.; Aebi M. Engineering *N*-linked protein glycosylation with diverse O antigen lipopolysaccharide structures in *Escherichia coli*. Proc. Natl. Acad. Sci. U. S. A. 2005, 102 (8), 3016–3021. 10.1073/pnas.0500044102. 15703289 PMC549450

[ref8] Valderrama-Rincon J. D.; Fisher A. C.; Merritt J. H.; Fan Y. Y.; Reading C. A.; Chhiba K.; Heiss C.; Azadi P.; Aebi M.; DeLisa M. P. An engineered eukaryotic protein glycosylation pathway in *Escherichia coli*. Nat. Chem. Biol. 2012, 8 (5), 434–436. 10.1038/nchembio.921. 22446837 PMC3449280

[ref9] Jaroentomeechai T.; Stark J. C.; Natarajan A.; Glasscock C. J.; Yates L. E.; Hsu K. J.; Mrksich M.; Jewett M. C.; DeLisa M. P. Single-pot glycoprotein biosynthesis using a cell-free transcription-translation system enriched with glycosylation machinery. Nat. Commun. 2018, 9 (1), 2686 10.1038/s41467-018-05110-x. 30002445 PMC6043479

[ref10] Natarajan A.; Jaroentomeechai T.; Cabrera-Sanchez M.; Mohammed J. C.; Cox E. C.; Young O.; Shajahan A.; Vilkhovoy M.; Vadhin S.; Varner J. D.; et al. Engineering orthogonal human O-linked glycoprotein biosynthesis in bacteria. Nat. Chem. Biol. 2020, 16 (10), 1062–1070. 10.1038/s41589-020-0595-9. 32719555 PMC7857696

[ref11] Hershewe J. M.; Warfel K. F.; Iyer S. M.; Peruzzi J. A.; Sullivan C. J.; Roth E. W.; DeLisa M. P.; Kamat N. P.; Jewett M. C. Improving cell-free glycoprotein synthesis by characterizing and enriching native membrane vesicles. Nat. Commun. 2021, 12 (1), 2363 10.1038/s41467-021-22329-3. 33888690 PMC8062659

[ref12] Stark J. C.; Jaroentomeechai T.; Moeller T. D.; Hershewe J. M.; Warfel K. F.; Moricz B. S.; Martini A. M.; Dubner R. S.; Hsu K. J.; Stevenson T. C. On-demand biomanufacturing of protective conjugate vaccines. Sci. Adv. 2021, 7 (6), eabe9444 10.1126/sciadv.abe9444. 33536221 PMC7857678

[ref13] aCelik E.; Fisher A. C.; Guarino C.; Mansell T. J.; DeLisa M. P. A filamentous phage display system for *N*-linked glycoproteins. Protein Sci. 2010, 19 (10), 2006–2013. 10.1002/pro.472. 20669235 PMC2998736

[ref14] Lipovsek D.; Pluckthun A. In-vitro protein evolution by ribosome display and mRNA display. J. Immunol. Methods 2004, 290 (1–2), 51–67. 10.1016/j.jim.2004.04.008. 15261571

[ref15] Guarino C.; DeLisa M. P. A prokaryote-based cell-free translation system that efficiently synthesizes glycoproteins. Glycobiology 2012, 22 (5), 596–601. 10.1093/glycob/cwr151. 22068020

[ref16] Tarui H.; Imanishi S.; Hara T. A novel cell-free translation/glycosylation system prepared from insect cells. J. Biosci. Bioeng. 2000, 90 (5), 508–514. 10.1016/S1389-1723(01)80031-1. 16232900

[ref17] aBuntru M.; Vogel S.; Stoff K.; Spiegel H.; Schillberg S. A versatile coupled cell-free transcription-translation system based on tobacco BY-2 cell lysates. Biotechnol. Bioeng. 2015, 112 (5), 867–878. 10.1002/bit.25502. 25421615

[ref18] Nakatogawa H.; Ito K. The ribosomal exit tunnel functions as a discriminating gate. Cell 2002, 108 (5), 629–636. 10.1016/S0092-8674(02)00649-9. 11893334

[ref19] Contreras-Martinez L. M.; DeLisa M. P. Intracellular ribosome display via SecM translation arrest as a selection for antibodies with enhanced cytosolic stability. J. Mol. Biol. 2007, 372 (2), 513–524. 10.1016/j.jmb.2007.06.070. 17669427

[ref20] Evans M. S.; Ugrinov K. G.; Frese M. A.; Clark P. L. Homogeneous stalled ribosome nascent chain complexes produced in vivo or in vitro. Nat. Methods 2005, 2 (10), 757–762. 10.1038/nmeth790. 16179922

[ref21] Schwarz F.; Lizak C.; Fan Y. Y.; Fleurkens S.; Kowarik M.; Aebi M. Relaxed acceptor site specificity of bacterial oligosaccharyltransferase in vivo. Glycobiology 2011, 21 (1), 45–54. 10.1093/glycob/cwq130. 20847188

[ref22] Martineau P.; Jones P.; Winter G. Expression of an antibody fragment at high levels in the bacterial cytoplasm. J. Mol. Biol. 1998, 280 (1), 117–127. 10.1006/jmbi.1998.1840. 9653035

[ref23] Juraja S. M.; Mulhern T. D.; Hudson P. J.; Hattarki M. K.; Carmichael J. A.; Nuttall S. D. Engineering of the *Escherichia coli* Im7 immunity protein as a loop display scaffold. Protein Eng., Des. Sel 2006, 19 (5), 231–244. 10.1093/protein/gzl005. 16549402

[ref24] Kocer I.; Cox E. C.; DeLisa M. P.; Celik E. Effects of variable domain orientation on anti-HER2 single-chain variable fragment antibody expressed in the *Escherichia coli* cytoplasm. Biotechnol. Prog. 2021, 37 (2), e3102 10.1002/btpr.3102. 33190426 PMC8842847

[ref25] Palmu A. A.; Jokinen J.; Borys D.; Nieminen H.; Ruokokoski E.; Siira L.; Puumalainen T.; Lommel P.; Hezareh M.; Moreira M.; et al. Effectiveness of the ten-valent pneumococcal *Haemophilus influenzae* protein D conjugate vaccine (PHiD-CV10) against invasive pneumococcal disease: a cluster randomised trial. Lancet 2013, 381 (9862), 214–222. 10.1016/S0140-6736(12)61854-6. 23158882

[ref26] Lu Z.; Madico G.; Roche M. I.; Wang Q.; Hui J. H.; Perkins H. M.; Zaia J.; Costello C. E.; Sharon J. Protective B-cell epitopes of *Francisella tularensis**O*-polysaccharide in a mouse model of respiratory tularaemia. Immunology 2012, 136 (3), 352–360. 10.1111/j.1365-2567.2012.03589.x. 22486311 PMC3385035

[ref27] Yen M.; Yin J. High-throughput profiling of posttranslational modification enzymes by phage display. Biotechniques 2007, 43 (1), 31–37. 10.2144/000112502. 17695643

[ref28] Arai K.; Tsutsumi H.; Mihara H. A monosaccharide-modified peptide phage library for screening of ligands to carbohydrate-binding proteins. Bioorg. Med. Chem. Lett. 2013, 23 (17), 4940–4943. 10.1016/j.bmcl.2013.06.059. 23871221

[ref29] Ng S.; Jafari M. R.; Matochko W. L.; Derda R. Quantitative synthesis of genetically encoded glycopeptide libraries displayed on M13 phage. ACS Chem. Biol. 2012, 7 (9), 1482–1487. 10.1021/cb300187t. 22725642

[ref30] Horiya S.; Bailey J. K.; Temme J. S.; Guillen Schlippe Y. V.; Krauss I. J. Directed evolution of multivalent glycopeptides tightly recognized by HIV antibody 2G12. J. Am. Chem. Soc. 2014, 136 (14), 5407–5415. 10.1021/ja500678v. 24645849 PMC4004241

[ref31] Ihssen J.; Kowarik M.; Wiesli L.; Reiss R.; Wacker M.; Thony-Meyer L. Structural insights from random mutagenesis of *Campylobacter jejuni* oligosaccharyltransferase PglB. BMC Biotechnol 2012, 12, 67 10.1186/1472-6750-12-67. 23006740 PMC3527161

[ref932] Cuccui J.; Thomas R. M.; Moule M. G.; D'Elia R. V.; Laws T. R.; Mills D. C.; Williamson D.; Atkins T. P.; Prior J. L.; Wren B. W. Exploitation of bacterial N-linked glycosylation to develop a novel recombinant glycoconjugate vaccine against Francisella tularensis. Open Biol. 2013, 3 (5), 130002 10.1098/rsob.130002. 23697804 PMC3866875

[ref32] Ollis A. A.; Chai Y.; Natarajan A.; Perregaux E.; Jaroentomeechai T.; Guarino C.; Smith J.; Zhang S.; DeLisa M. P. Substitute sweeteners: diverse bacterial oligosaccharyltransferases with unique N-glycosylation site preferences. Sci. Rep 2015, 5, 15237 10.1038/srep15237. 26482295 PMC4894442

[ref33] Jewett M. C.; Swartz J. R. Mimicking the *Escherichia coli* cytoplasmic environment activates long-lived and efficient cell-free protein synthesis. Biotechnol. Bioeng. 2004, 86 (1), 19–26. 10.1002/bit.20026. 15007837

