# Supplementary material for: Ribosome Stalling of N-Linked Glycoproteins in Cell-Free Extracts
Source: ACS Synth Biol. 2022 Nov 18;11(12):3892–9. doi: 10.1021/acssynbio.2c00311 (PMC9764415; doi:10.1021/acssynbio.2c00311)
Supplement: Supplementary file 1 — sb2c00311_si_001.pdf [file sb2c00311_si_001.pdf]

Supplementary Information

Ribosome display of *N*-linked glycoproteins in cell-free extracts

Sean S. Chung[1], Erik J. Bidstrup[2], Jasmine M. Hershewe[3,4,5], Katherine F. Warfel[3,4,5], Michael C. Jewett[3,4,5] and Matthew P. DeLisa[1,2,6]*

[1]Biochemistry, Molecular and Cell Biology, Cornell University, Ithaca, NY 14853 USA
[2]Robert F. Smith School of Chemical and Biomolecular Engineering, Cornell University, Ithaca, NY 14853 USA
[3]Department of Chemical and Biological Engineering, Northwestern University, 2145 Sheridan Road Technological Institute E136, Evanston, IL USA, 60208-3120
[4]Center for Synthetic Biology, Northwestern University, 2145 Sheridan Road Technological Institute E136, Evanston, IL USA, 60208-3120
[5]Chemistry of Life Processes Institute, Northwestern University, 2170 Campus Drive, Evanston, IL USA, 60208-3120
[6]Cornell Institute of Biotechnology, Cornell University, Ithaca, NY 14853 USA

*Address correspondence to: Matthew P. DeLisa, Robert Frederick Smith School of Chemical and Biomolecular Engineering, Cornell University, Ithaca, NY 14853. Tel: 607-254-8560; Email: md255@cornell.edu

Supplementary Methods

Plasmids. All plasmids constructed in this study were made using standard cloning protocols. For *in vivo* expression, plasmid pET–Im7^{N58}–SecM17 was constructed by inserting the PCR-amplified product encoding Im7^{N58} in place of the gene encoding scFv13–R4 in plasmid pET–scFv13–R4–SecM17 [1]. Plasmid pET–^{DQNAT}scFv13–R4–SecM17 was constructed by similar replacement but with a PCR-amplified product encoding ^{DQNAT}scFv13-R4 in which the N-terminal DQNAT motif was introduced via the forward primer. Plasmid pET–Im7^{N58} was constructed by PCR amplification of the gene encoding Im7^{N58} from plasmid pTrc99S–YebF–Im7^{N58} [2] and subsequent Gibson isothermal assembly [3] of the resulting PCR product into the amplified plasmid backbone of pET28a. For *in vitro* expression, plasmid pJL1–Im7^{N58}–SecM17 was constructed by PCR amplification of the gene encoding Im7^{N58}–SecM17 from plasmid pET–Im7^{N58}–SecM17 and Gibson assembly of the resulting PCR product into the amplified pJL1 plasmid backbone. Plasmids pJL1–scFv–HER2^{DQNAT}–SecM17 and pJL1–^{DQNAT}PD–SecM17 were constructed by PCR amplification of the genes encoding scFv–HER2^{DQNAT} from plasmid pTrc99S–YebF–scFv–HER2^{DQNAT} [2] and PD from plasmid pJL1–PD^{4xDQNAT} [4], respectively, and inserting the resulting PCR products in place of Im7^{N58} in amplified plasmid pJL1–Im7^{N58}–SecM17 via Gibson assembly. The same PCR products were used to construct plasmids pJL1–scFv–HER2^{DQNAT} and pJL1–^{DQNAT}PD by Gibson assembly into plasmid pJL1. Additional plasmids constructed previously and used in this study included: plasmid pSN18 for expression and purification of *Cj*PglB [5]; plasmid pSF–*Cj*PglB for selective enrichment of *Cj*PglB in cell-free extracts [6]; plasmid pMW07–pglΔB for expression and extraction of *Cj*LLOs [7]; and plasmid pET28–ColE7^{H569A} for expression and purification of ColE7 [2]. All plasmids were confirmed by DNA sequencing at the Biotechnology Resource Center (BRC) Genomics Facility (RRID:SCR_021727) in the Cornell Institute of Biotechnology.

Ribosome isolation. Cells transformed with the pET28a-derived constructs were grown in 100-mL cultures and induced with 1 mM IPTG at an Abs_{600} ~0.5 and grown at 30 °C for an additional 30 min. Following expression, two buffer C (20 mMTris–HCl (pH 7.5), 50 mM NH_4Cl, 25 mM $MgCl_2$) ice cubes were added to each culture flask, rapidly shaken for 1 min on ice and incubated on ice for an additional 30 min. Next, cells were pelleted by

centrifugation for 30 min at 4 °C and 10,000×g and resuspended in 600 μL of cold buffer C. Cells were lysed by three cycles of freeze–thawing in liquid nitrogen followed by the addition of three 30-μL aliquots of lysozyme (Novagen), where the stock lysozyme solution was diluted 50-fold in cold buffer C and each lysozyme addition was followed by a 20 min incubation at 4 °C, and finally three additional freeze–thawing cycles. Ribosomes were isolated from these soluble fractions according to previously published procedures [1, 8]. Specifically, three 12-μl aliquots of RQ1DNase (Promega) were added to reduce the viscosity of the lysates and samples were rotated for 15 min at 4 °C after each dose of the enzyme. Samples were spun in a microcentrifuge for 20 min at 10,000×g at 4 °C to pellet the debris. To isolate ribosomes, the supernatant was collected and loaded onto a cold cushion made up of equal volumes of buffer C supplemented with a 5% (w/v) sucrose phase and buffer B (20 mM Tris–HCl (pH 7.5), 500 mM NH_4Cl, 25 mM $MgCl_2$) supplemented with a 37% sucrose phase. Ribosomes were isolated by ultracentrifugation for 35 h at 120,000×g and 4 °C using a Beckman LS 8 ultracentrifuge with an SW28 rotor. The crude ribosome pellet was resuspended in 200 μL of buffer C and ultracentrifuged in a 10% to 40% (w/v) sucrose gradient in buffer A (20 nM Tris–HCl (pH 7.5), 100 mM NH_4Cl, 25 mM $MgCl_2$) for 17 h at 71,000×g and 4 °C in a SW41 rotor. Gradient fractionation was performed manually by pipetting 250 μL at a time from the top part of the gradient. All collected samples were stored at 4 °C for further analysis. A similar procedure was followed for isolation of ribosomes from cell-free extracts.

Expression and purification of *Cj*PglB. Briefly, a single colony of *E. coli* CLM24 carrying plasmid pSN18 [5] was grown overnight at 37 °C in 50 mL of LB (10 g/L tryptone, 5 g/L yeast extract, 5 g/L NaCl, pH 7.2) supplemented with ampicillin (Amp) and 0.2% (w/v) D-glucose. Overnight cells were sub-cultured into 1 L of fresh terrific broth (TB; 12 g/L tryptone, 24 g/L yeast extract, 0.4% (v/v) glycerol, 10% (v/v) 0.17 M KH_2PO_4/0.72 M K_2HPO_4 phosphate buffer), supplemented with Amp and grown until Abs_{600} reached a value of ~0.7. The incubation temperature was adjusted to 16 °C, after which protein expression was induced by the addition of L-arabinose to a final concentration of 0.02% (w/v). Protein expression was allowed to proceed for 20 h at 16 °C. Cells were harvested by centrifugation and then disrupted using a homogenizer (Avestin C5 EmulsiFlex). The lysate was centrifuged to remove cell debris and the supernatant was ultracentrifuged

(100,000×g) for 2 h at 4 °C. The resulting pellet containing the membrane fraction was fully resuspended with a Potter-Elvehjem tissue homogenizer in buffer containing 50 mM HEPES, 250 mM NaCl, 10% (v/v) glycerol, and 1% (w/v) n-dodecyl-β-D-maltoside (DDM) at pH 7.5. The suspension was incubated at room temperature for 1 h to facilitate detergent solubilization of *Cj*PglB from native *E. coli* lipids, which were removed by subsequent ultracentrifugation (100,000×g) for 1 h at 4 °C. The supernatant containing DDM-solubilized *Cj*PglB was purified using Ni-NTA resin (ThermoFisher) according to manufacturer's specification with the exception that all buffers were supplemented with 1% (w/v) DDM. The elution fraction from Ni-NTA purification was then subjected to size exclusion chromatography (SEC) using an ÄKTA Explorer FPLC system (GE Healthcare) with Superdex 200 10/300 GL column. Purified protein was stored at a final concentration of 1-2 mg/mL in OST storage buffer (50 mM HEPES, 100 mM NaCl, 5% (v/v) glycerol, 0.01% (w/v) DDM, pH 7.5) at 4 °C. Glycerol concentration in the sample was adjusted to 20% (v/v) for long-term storage at -80 °C.

Expression and purification of ColE7. To produce ColE7 for ELISA and affinity enrichment experiments, an overnight culture BL21(DE3) cells carrying plasmid pET28a-ColE7^{H569A} was used to inoculate 1 L of LB supplemented with 50 µg/mL kanamycin. Cells were grown at 37 °C until mid-log phase and then were induced with 0.1 mM IPTG for 16 h at 16 °C before being harvested. Following centrifugation at 10,000×g, pellets were resuspended in PBS buffer supplemented with 10-mM imidazole and lysed at 16,000–18,000 psi using an Emulsiflex-C5 homogenizer (Avestin). The lysate was clarified by centrifugation at 15,000×g for 30 min at 4 °C and the collected soluble fraction was mixed with Ni-NTA resin for 2 h at 4 °C. The mixture was then applied to a gravity flow column and washed with 5 column volumes of PBS containing 20 mM imidazole. Proteins were eluted in 4 column volumes of PBS with 250-mM imidazole. The eluted protein was desalted and concentrated to 5 mg/mL in PBS buffer using Ultracentrifugal Filters with 10-kDa molecular weight cut-off (Amicon) and stored at 4 °C.

Extraction of LLOs. The protocol for organic solvent extraction of LLOs from *E. coli* membranes was adapted from a previously described protocol [9]. Briefly, a single colony of strain CLM24 carrying plasmid pMW07-pglΔB for expression of the *C. jejuni N*-glycan on undecaprenylphophate was grown overnight in LB media. Overnight cells were sub-

cultured into 1.0 L of TB supplemented with chloramphenicol and grown until the Abs_{600} reached ~0.7. The incubation temperature was adjusted to 30 °C and protein expression was induced with L-arabinose at a final concentration of 0.2% (w/v). After 16 h, cells were harvested by centrifugation and cell pellets were lyophilized to complete dryness at -70 °C using methanol. For extraction of *Cj*LLOs, the lyophilisates were resuspended in 12 mL of 2:1 (v/v) $CHCl_3$:CH_3OH and sonicated at a 1:1 duty cycle, 5 s cycles, for 100 s. Samples were then centrifuged for 10 min at 3,000×g and the supernatant was decanted, retaining the pellet. The resuspension, sonication, centrifugation, and decanting were then repeated with that pellet. The pellet was then resuspended by vortexing in 20 mL H_2O, sonicated for 4–5 min until homogenous, centrifuged, and decanted once more. Pellets were then resuspended in 10:20:3 (v/v/v) $CHCl_3$:CH_3OH:H_2O solution, sonicated until homogenous, and incubated at room temperature for 15 min to facilitate extraction of LLOs. 8 mL of methanol were then added, and samples were centrifuged as before, saving the supernatant while discarding the pellet. The supernatant then had 10 mL of 4:1 (v/v) of chloroform/H_2O added and was then centrifuged (4,000×g) for 15 min, after which the organic layer (bottom layer, including flakes) was collected and dried with a vacuum concentrator followed by lyophilization. All lyophilisates containing active *Cj*LLOs were resuspended in cell-free glycosylation buffer (10 mM HEPES, pH 7.5, 10 mM $MnCl_2$, and 0.1% (w/v) DDM) and stored at 4 °C.

Western blot analysis. To concentrate ribosome fractions for Western blot analysis, volumes of the collected ribosomal fractions were mixed with cold 20% (v/v) trichloroacetic acid (TCA) in a 1:2 volume ratio and allowed to precipitate for 30 min on ice. After ultracentrifugation for 20 min at 210,000 and 4 °C, the pellet was dried of all remaining TCA and directly resuspended in SDS–PAGE loading buffer. Ribosome fractions were resolved by SDS-polyacrylamide gel electrophoresis on 10% Mini-PROTEAN TGX™ Precast Protein Gels (Bio-Rad). Prior to loading, fractions were normalized by rRNA content as measured by A260 (see **Supplementary Figs. 1** and **2**). For visualizing the separated protein samples, gels were stained with Coomassie G-250 stain (Bio-Rad) following the manufacturer's protocol. For Western blot analysis, the separated protein samples were then transferred to nitrocellulose membranes using a semi-dry apparatus. Following transfer, the membranes were blocked with 5% milk (w/v)

in TBST (1x TBS, 0.1% Tween 20) and subsequently probed for 1 h with one of the following: horseradish peroxidase (HRP)-conjugated anti-DDDDK antibody (Abcam, cat # ab49763) that recognized the FLAG epitope tag; the *C. jejuni* heptasaccharide glycan-specific antiserum hR6 (kindly provided by Markus Aebi); or the mouse mAb FB11 (ThermoFisher; cat # MA1-7388) that specifically recognizes *F. tularensis* LPS. Goat anti-rabbit IgG (HRP) (Abcam, cat # ab205718) was used as the secondary antibody to detect hR6 antiserum while HRP-conjugated anti-mouse IgG (Abcam, cat # ab97023) was used as the secondary antibody to detect FB11. After washing five to six times with TBST for 5 min, the membranes were visualized using a ChemiDoc MP Imaging System (Bio-Rad).

ELISA. Binding activity for ribosome-tethered Im7^{N58} and scFv–HER2^{DQNAT} was determined by standard ELISA. Briefly, treated Costar 96-well ELISA plates (Corning) were coated overnight at 4 °C with 50 μL of 5-μg/mL in-house prepared ColE7 in PBS for ribosome-tethered Im7^{N58} or the same amount of commercial extracellular domain (residues 1 to 652) of human HER2 (HER2-ED; Sino Biological, 10004-HCCH) for ribosome-tethered scFv–HER2^{DQNAT} in PBS. After blocking with 200 μL of blocking solution (1% (w/v) non-fat milk, 5 mM $MgCl_2$, 2.5 mg/ml of heparin, 0.05 mg/ml *E. coli* tRNA in PBS), for 2 h at room temperature, the plates were washed four times with washing solution (0.1% (v/v) Tween 20, 5 mM $MgCl_2$ in PBS). Isolated 70 S ribosome samples displaying Im7^{N58} and scFv–HER2^{DQNAT} were mixed gently with equal volume of cold blocking solution, and 100 μl of this mixture was added to each well. The plate was incubated for 1 h at 4 °C and then washed five to six times with 200 μl of cold washing solution at 4 °C to remove any unbound complexes. After washing, 50 μL of the HRP-conjugated anti-DDDK antibody (see above) in 1% PBST was added to each well for 1 h. Plates were washed three times and then developed using 50 μL 1-Step Ultra TMB-ELISA substrate solution (ThermoFisher). A similar ELISA protocol was followed for detecting RNC complexes displaying glycosylated ^{DQNAT}PD except that the plates were coated with 50 μL of 5-μg/mL mouse mAb FB11 in PBS and were subsequently detected with HRP-conjugated anti-mouse IgG.

Statistical analysis and reproducibility. To ensure robust reproducibility of all results, experiments were performed with multiple biological replicates and technical measurements. Sample sizes were not predetermined based on statistical methods but

were chosen according to the standards of the field (at least three independent biological replicates for each condition), which gave sufficient statistics for the effect sizes of interest. All data were reported as average values with error bars representing standard deviation. Statistical significance was determined by unpaired *t* test with Welch's correction (*$p < 0.05$, **$p < 0.01$; ns, not significant). All graphs were generated using Prism 9 for MacOS version 9.2.0. No data were excluded from the analyses. The experiments were not randomized. The Investigators were not blinded to allocation during experiments and outcome assessment.

References Cited

(1) Contreras-Martinez, L. M.; DeLisa, M. P. Intracellular ribosome display via SecM translation arrest as a selection for antibodies with enhanced cytosolic stability. *J Mol Biol* **2007**, *372* (2), 513-524.

(2) Li, M.; Zheng, X.; Shanker, S.; Jaroentomeechai, T.; Moeller, T. D.; Hulbert, S. W.; Kocer, I.; Byrne, J.; Cox, E. C.; Fu, Q.; et al. Shotgun scanning glycomutagenesis: A simple and efficient strategy for constructing and characterizing neoglycoproteins. *Proc Natl Acad Sci U S A* **2021**, *118* (39).

(3) Gibson, D. G.; Young, L.; Chuang, R. Y.; Venter, J. C.; Hutchison, C. A., 3rd; Smith, H. O. Enzymatic assembly of DNA molecules up to several hundred kilobases. *Nat Methods* **2009**, *6* (5), 343-345.

(4) Stark, J. C.; Jaroentomeechai, T.; Moeller, T. D.; Hershewe, J. M.; Warfel, K. F.; Moricz, B. S.; Martini, A. M.; Dubner, R. S.; Hsu, K. J.; Stevenson, T. C.; et al. On-demand biomanufacturing of protective conjugate vaccines. *Sci Adv* **2021**, *7* (6).

(5) Kowarik, M.; Numao, S.; Feldman, M. F.; Schulz, B. L.; Callewaert, N.; Kiermaier, E.; Catrein, I.; Aebi, M. N-linked glycosylation of folded proteins by the bacterial oligosaccharyltransferase. *Science* **2006**, *314* (5802), 1148-1150.

(6) Ollis, A. A.; Chai, Y.; Natarajan, A.; Perregaux, E.; Jaroentomeechai, T.; Guarino, C.; Smith, J.; Zhang, S.; DeLisa, M. P. Substitute sweeteners: diverse bacterial oligosaccharyltransferases with unique N-glycosylation site preferences. *Sci Rep* **2015**, *5*, 15237.

(7) Ollis, A. A.; Zhang, S.; Fisher, A. C.; DeLisa, M. P. Engineered oligosaccharyltransferases with greatly relaxed acceptor-site specificity. *Nat Chem Biol* **2014**, *10* (10), 816-822.

(8) Evans, M. S.; Ugrinov, K. G.; Frese, M. A.; Clark, P. L. Homogeneous stalled ribosome nascent chain complexes produced in vivo or in vitro. *Nat Methods* **2005**, *2* (10), 757-762.

(9) Guarino, C.; DeLisa, M. P. A prokaryote-based cell-free translation system that efficiently synthesizes glycoproteins. *Glycobiology* **2012**, *22* (5), 596-601.

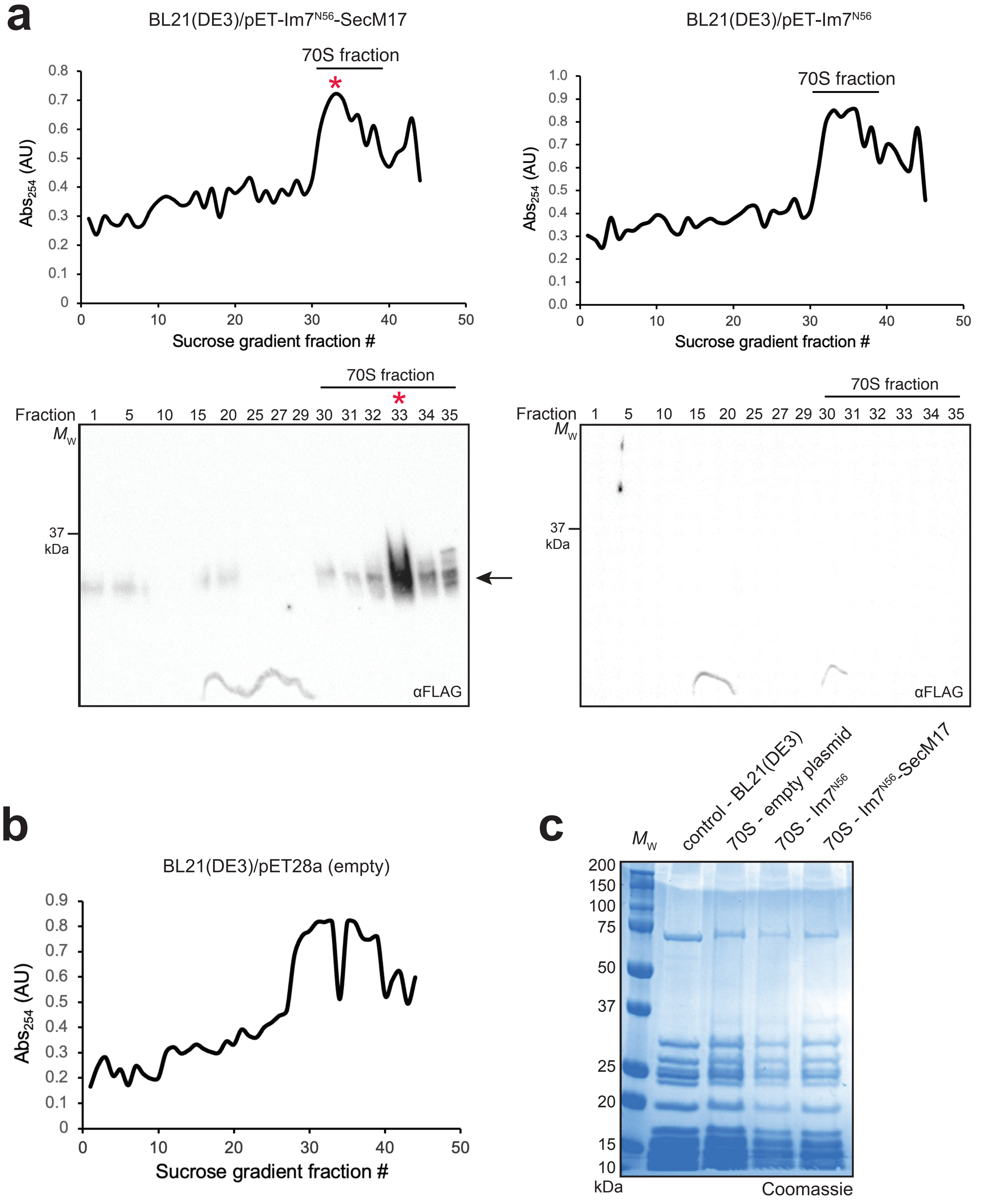

Supplementary Figure 1. Purification and characterization of 70S ribosomes displaying Im7^{N56}-SecM17. (a, top) Absorbance trace (254 nm) of sucrose fractions derived from BL21(DE3) cells expressing Im7^{N56}-SecM17. Red asterisk indicates the fraction selected for further downstream analysis and used in hybrid glycoRNC reactions. (a, bottom) Western blot analysis of selected 70S ribosome sucrose fractions from (a, top) as indicated. Blots were probed with α-FLAG-HRP antibody specific for epitope tag on Im7^{N56}-SecM17. Black arrow indicates expected molecular weight of Im7^{N56}-SecM17. (b) Absorbance trace (254 nm) of sucrose fractions derived from BL21(DE3) cells carrying empty pET28a plasmid. (c) Coomassie-stained SDS-PAGE gel of sucrose fractions enriched with 70S ribosomes from BL21(DE3) cells without a plasmid, with empty pET28a plasmid, expressing Im7^{N56}, and expressing Im7^{N56}-SecM17. Molecular weight (M_W) markers are indicated on left of Western blots and SDS-PAGE gel. All results are representative of biological replicates (n = 2).

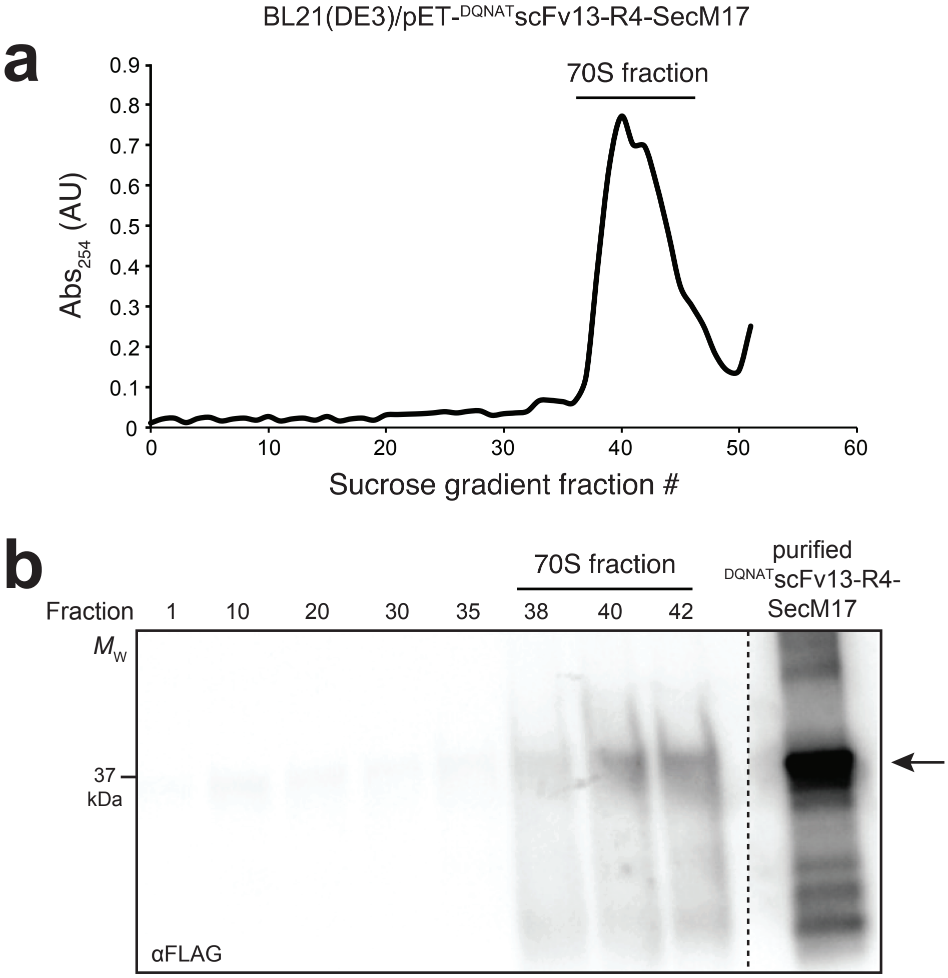

Supplementary Figure 2. Purification and characterization of 70S ribosomes displaying ^{DQNAT}scFv13-R4-SecM17. (a) Absorbance trace (254 nm) of sucrose fractions derived from BL21(DE3) cells expressing ^{DQNAT}scFv13-R4-SecM17. Results are representative of biological replicates (n = 2). (b) Western blot analysis of selected 70S ribosome sucrose fractions from (a) as indicated. Blots were probed with α-FLAG-HRP antibody specific for epitope tag on ^{DQNAT}scFv13-R4-SecM17. Black arrow indicates the expected molecular weight of ^{DQNAT}scFv13-R4-SecM17, as demonstrated by the purified control at right. Molecular weight (M_{W}) markers are indicated on left. Western blot is representative of biological replicates (n = 2).
